# Interaction between Soluble and Membrane-Embedded Potassium Channel Peptides Monitored by Fourier Transform Infrared Spectroscopy

**DOI:** 10.1371/journal.pone.0049070

**Published:** 2012-11-08

**Authors:** Geoffrey W. Abbott, Bala Ramesh, Surjit K. Srai

**Affiliations:** 1 Department of Pharmacology, Department of Physiology and Biophysics, School of Medicine, University of California Irvine, Irvine, California, United States of America; 2 Department of Biochemistry and Molecular Biology, University College London, London, United Kingdom; Max Planck Institute for Polymer Research, Germany

## Abstract

Recent studies have explored the utility of Fourier transform infrared spectroscopy (FTIR) in dynamic monitoring of soluble protein-protein interactions. Here, we investigated the applicability of FTIR to detect interaction between synthetic soluble and phospholipid-embedded peptides corresponding to, respectively, a voltage-gated potassium (Kv) channel inactivation domain (ID) and S4–S6 of the *Shaker* Kv channel (KV1; including the S4–S5 linker “pre-inactivation” ID binding site). KV1 was predominantly α-helical at 30°C when incorporated into dimyristoyl-l-α-phosphatidylcholine (DMPC) bilayers. Cooling to induce a shift in DMPC from liquid crystalline to gel phase reversibly decreased KV1 helicity, and was previously shown to partially extrude a synthetic S4 peptide. While no interaction was detected in liquid crystalline DMPC, upon cooling to induce the DMPC gel phase a reversible amide I peak (1633 cm^−1^) consistent with novel hydrogen bond formation was detected. This spectral shift was not observed for KV1 in the absence of ID (or vice versa), nor when the non-inactivating mutant V7E ID was applied to KV1 under similar conditions. Alteration of salt or redox conditions affected KV1-ID hydrogen bonding in a manner suggesting electrostatic KV1-ID interaction favored by a hairpin conformation for the ID and requiring extrusion of one or more KV1 domains from DMPC, consistent with ID binding to S4–S5. These findings support the utility of FTIR in detecting reversible interactions between soluble and membrane-embedded proteins, with lipid state-sensitivity of the conformation of the latter facilitating control of the interaction.

## Introduction

Despite recent dramatic advances in membrane protein crystallography and other structural techniques, development of systems in which dynamic protein-protein interactions can be detected and studied is still warranted. While Fourier transform infrared spectroscopy (FTIR) is a relatively low resolution technique in terms of structural information compared to e.g., X-ray crystallography, FTIR can detect changes in protein conformation, or interaction via novel hydrogen-bonding, via spectral shifts in the amide I region [1], [2]. In addition, FTIR offers advantages including dynamic, nondestructive monitoring of protein samples, and ease of use in a wide range of protein states and environments. Previous FTIR studies showed that the S4 voltage-sensing segment of voltage-gated potassium (Kv) channels adopts an α-helical conformation when incorporated into lipid, and that phase transition shifts from the liquid crystalline (LC) phase to the gel phase could be used to reversibly extrude S4 from the lipid [3].

Some Kv channels include a cytoplasmic inactivation domain (ID) that facilitates rapid “N-type” channel inactivation following voltage-dependent activation of the channel. The ID is a cytoplasmic tethered “ball” that can bind to the intracellular S4–S5 linker region following depolarization-initiated activation of S4, probably resulting in a shift in the Kv channel to a “pre-inactivated” state [4]–[6]. This pre-inactivated state is thought to be both voltage-independent (because it occurs at a binding site outside the membrane electric field), and the rate-limiting step of N-type inactivation. Following this pre-inactivation step, the ID can move further into the channel pore to a deeper, hydrophobic site at which it occludes K^+^ movement through the pore despite the channel being open – hence, inactivation [6].

Kv channels comprise tetramers of six transmembrane (TM)-domain α subunits, each with a single P-loop region and extensive N- and C-terminal cytoplasmic domains [7]. While the deep pore binding of the ID is expected to require a fully-folded, tetrameric Kv channel to provide the appropriate pore conformation, the pre-inactivation step is thought to involve 1∶1 binding between the ID and any one of the four S4–S5 linkers in a tetrameric Kv channel. Extensive studies have shown that synthetic peptide corresponding to the ID is capable of channel inactivation in Kv channels with their intrinsic ID removed, or in non-ID channels. Also, a number of studies investigating the fast-inactivation process at a structural level have employed synthetic ID peptides and model targets such as anionic lipids which are proposed to approximate a negatively-charged outer “pre-inactivation” ID-binding site and an inner, hydrophobic ID-binding pocket [8]–[11].

Synthetic S4 peptide was previously found to be extruded by a temperature-induced lipid phase transition in a manner suggested to mimic S4 activation, which occurs prior to pre-inactivation [3]. Also, X-ray crystallography studies have shown adoption of ordered, putatively native, structure, by monomeric voltage sensor paddles reconstituted into lipid; furthermore, voltage sensor conformation and response to sensor-binding toxins are highly sensitive to the mechanical state of the lipids in which they are embedded [12]–[15].

Here, interested in examining the utility of FTIR in detecting dynamic interactions between peptides/proteins, we synthesized a polypeptide (KV1) comprising the S4–S6 (including S5 and the S4–S5 linker) region of *Shaker* Kv channel and analyzed its interaction with a synthetic Kv3.4 ID peptide in response to temperature-controlled lipid phase shifts. We detected, using FTIR, reversible spectral changes consistent with intermolecular KV1-ID hydrogen bonding upon liquid crystal to gel phase transition of the KV1-embedding lipid. Detection of the interaction, specificity of which was suggested by sensitivity to mutation and environment, is proof-of-principle of the utility of this system for probing dynamic interactions between soluble and membrane-embedded proteins.

## Methods

### Peptide Synthesis and Preparation

**Figure 1:**
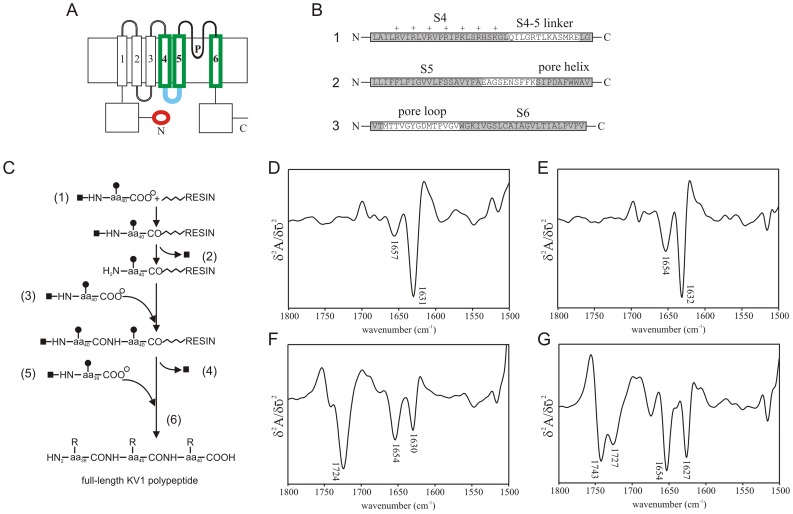
Synthesis and FTIR spectra of KV1 polypeptide. A. Membrane-spanning topology of *Shaker* Kv channel. Colored regions denote domains within the KV1 polypeptide (green, S4, S5 and S6; blue, S4–S5 linker) and the ID peptide (red). B. The three short peptides (numbered 1, 2 & 3) joined by solid-phase fragment condensation synthesis to construct the 119-residue KV1 polypeptide. Primary structure, proposed structural/functional domains and S4 positive charges are indicated. C. Schematic representation of solid phase fragment condensation synthesis of the KV1 polypeptide: (1) First protected 40-residue fragment is assembled on solid support; (2) α-amino group is deprotected; (3) Second protected 40-residue fragment reacts; (4) Deprotection is repeated; (5) Third protected 39-residue fragment reacts (6) Full-length KV1 polypeptide is deprotected and cleaved from support. **▪** = protecting group; **•** = side chain protecting group; **Λ** = linker to solid support. D. Second derivative FTIR spectrum of KV1 polypeptide (10 mg/ml) in 25 mM SDS at 30°C. Peptide was solubilized by simple mixing with SDS in deuterated PBS. E. Second derivative FTIR spectrum of KV1 polypeptide (10 mg/ml) in 25 mM SDS at 30°C. Peptide was prepared as a thin-film, then reconstituted by adding a thin film of SDS, before solubilization in deuterated PBS. F. Second derivative FTIR spectrum of KV1 polypeptide (10 mg/ml) in 50 mg/ml lysophosphatidylcholine (LPC) at 30°C. Peptide was prepared as a thin-film, then reconstituted by adding a thin film of LPC, before solubilization in deuterated PBS. G. Second derivative FTIR spectrum of KV1 polypeptide (10 mg/ml) in 50 mg/ml DMPC at 30°C. Peptide was prepared as a thin-film, then reconstituted by adding a thin film of DMPC, before solubilization in deuterated PBS.

A synthetic 119-residue polypeptide corresponding to the *Shaker* B Kv α subunit S4/S5/P-loop/S6 region (Fig. 1A, B) [16], hereafter referred to as ‘KV1’, was prepared for structural analysis. The synthesis of KV1 was achieved by solid-phase fragment condensation synthesis of three peptides, two of 40 residues and one of 39 residues, using a Rainin PS3 automated peptide synthesizer (Protein Technologies, Inc., Woburn, MA, U.S.A.) by a step-wise solid-phase procedure [17] using α-9-fluorenylmethyloxycarbonyl (Fmoc) protecting group strategy (Fig. 1C), as follows:

The sequence of 40 amino acids beginning at the carboxyl terminal of the S6 TM region was assembled first onto Rink-amide resin. The Rink-amide-MBHA resin (0.5 g) functionalized with Fmoc-amide was placed in a glass reaction vessel and deprotection carried out using 20% piperidine in DMF. After removal of the Fmoc group on the resin, acylation with the first amino acid active esters prepared from Fmoc-amino acid TBTU was carried out. A 5-fold excess (based on the resin loading) of acylating species in 0.2 M MMP in DMF (5 ml) was used throughout the synthesis. The synthesis was performed using the batch method on an automated peptide synthesizer with standard protocols and scale (0.1 mmol theoretical yield of crude peptide). On completion of synthesis, this first protected peptide fragment was left attached to the resin for the subsequent fragment condensation synthesis.

To generate fully protected polypeptide fragments containing the respective TM and P-loop regions, the next two polypeptides (40 and 39 residues, respectively) beginning one after the end of the previous fragment were synthesized as for the first fragment. Hyper acid-labile linker (HMPB)-containing resin was used in this phase of the synthesis to generate fully protected polypeptide fragments. On completion of synthesis of the respective polypeptide fragments, the polypeptide-containing HMPB-resins were pre-swelled in dichloromethane (DCM) in sintered glass columns and the excess DCM removed by vacuum. To detach the protected polypeptides, cleavage solution containing 1% TFA in DCM (10 ml) was added to sintered glass columns and agitated with nitrogen for 3 minutes. Cleavage solutions containing the protected polypeptides were filtered into round bottomed flasks containing 10% pyridine in methanol (5 ml) and rotary evaporated to quarter the original volume. Cold diethyl ether (40 ml) was added to the filtrate to aid precipitation of the product, and for repeated wash steps, before drying the samples under high vacuum in a freeze-drier.

The dried, protected polypeptides were then applied step-wise as activated active esters to the deprotected first protected fragment still attached to the Rink-amide resin. The coupling reaction was allowed to proceed for 30 minutes after which the resin was washed with DMF. The resin was then subjected to a final deprotection step to remove the N^α^-Fmoc of the second fragment. Finally the third activated protected fragment was added to the resin and allowed to react for 30 minutes. After synthesis, the N^α^-Fmoc-protective group of the 119-residue KV1 polypeptide was removed using 20% piperidine in DMF. The exposed N^α^ group was blocked by acetylation by adding Ac_2_O to the aminopeptidyl-resin. After acetylation the polystyrene resins were resuspended in 10% TFA in DCM and agitated with nitrogen in a sintered column, and collected at intervals. Following this, the TFA and DCM were completely removed by rotary evaporation and the protected polypeptide amides concentrated. Finally, to completely remove all the protecting groups, 82% TFA in the presence of scavengers thioanisole (5%), phenol (5%) and ethanedithiol were added to the residual oily solution and left at room temperature with occasional swirling for 4 hours. The TFA and scavengers were removed by rotary evaporation at 50°C. The crude products were precipitated and thoroughly washed with chilled anhydrous diethyl ether, then dried under high vacuum.

Crude KV1 polypeptide was solubilized in TFA/TFE (1∶1, v/v), injected into a Vydac C4 (214 TP 1010 RP preparative) column, equilibrated in 50% buffer A (TFA/CH_3_CN/H2O 1∶1∶98) and 50% buffer B (TFA/CH_3_CN/H2O 1∶90∶9), and purified on a linear gradient of buffer B with multiple reverse-phase HPLC (Varian, USA) runs. Sample homogeneity was confirmed by further HPLC analysis on a narrow-bore Vydac C4 (214 TP 54 RP analytical) column. Following purification, one major peak was observed when the eluents were monitored at 280 nm; analysis by SDS-PAGE gave a single band at an appropriate migration distance. While laser mass analysis was not possible due to solubilization difficulties, identity and purity were further confirmed by amino acid analysis (Table 1). The fraction containing pure peptide was lyophilized in the presence of 0.1 M HCl to remove TFA in preparation for structural studies.

**Table 1:** Amino acid analysis of synthetic KV1 polypeptide.

Amino acid	Actual	Expected
Asx	2.85	3
Leu	14.01	14
Ile	10.97	11
Arg	7.03	7
Val	15.00	15
Phe	10.95	11
Lys	5.00	5
Ser*	8.02	9
His	1.03	1
Gly	10.98	11
Glx	3.97	4
Ala	8.85	9
Met*	2.56	3
Pro	4.00	4
Trp†	–	3
Tyr	1.97	2
Cys	0.87	1

Values shown are actual proportions of each residue compared to numbers expected from the primary sequence.

* Ser and Met partially destroyed by hydrolysis;

† Trp completely destroyed by hydrolysis.

Reconstitution of the highly hydrophobic KV1 polypeptide into detergent or lipid for examination of its conformation in membrane-mimicking environments was performed either by simple mixing of polypeptide with lipid or detergent in buffered saline with 0–40% trifluoroethanol (TFE), or by one of 2 thin-film reconstitution techniques.

The first method, in cases where TFE was not used in the sample buffer, involved formation of a thin film of peptide onto the wall of a round-bottomed flask by rotary evaporation of a solution of the peptide in TFA. Following salting-out of the TFA using 0.1 mM HCl and subsequent freeze-drying, a thin film of lipid or detergent (solubilized in chloroform/methanol) was applied over the peptide by rotary evaporation; the double thin-film was then taken up into PBS prepared with deuterated water (DBS).

The second method, used for DMPC-TFE sample buffers, was by a TFE-based ‘thin-film’ lipid/detergent reconstitution technique as previously described [18]. Briefly, this involved formation of a thin film of peptide solubilized in TFE. Once all the TFE was evaporated off, a solution containing the appropriate concentration of DMPC solubilized in TFE was applied to the thin peptide film to achieve co-solubilization of the peptide with the DMPC in TFE. After this, DBS was gradually added to the peptide/DMPC/TFE solution until the final composition of the mixture was 10–40% TFE (v/v) (as stated in the text), 10 mg/ml KV1 polypeptide and 25–100 mg/ml DMPC (as stated in the text) in DBS (pH 7.4). Conformation of KV1 polypeptide in all experimental conditions was analyzed using FTIR spectroscopy to assess the effect on conformation of a range of environments, and before addition of ID peptide so that effect of subsequent addition of ID peptide could be assessed.

Wild-type (WT) [19] or V7E mutant [8] Kv3.4 ID peptides, sequence: MISSVC*V/E*SSYRGRKSGNKPPSKTCLKEE (corresponding to the first 28 residues of Kv3.4) were synthesized using a Rainin PS3 automated peptide synthesizer (Protein Technologies, Woburn, MA, U.S.A.) by a step-wise solid-phase procedure using Fmoc protecting group strategy, purified and verified by mass spectrometry as previously described [8]. Infrared spectra of the ID peptides in all experimental conditions were recorded in order in order to assess their inherent contribution to experimental spectra arising from ID/KV1 interaction. Oxidation or reduction of ID peptides was performed as previously described [8] prior to mixing with KV1 polypeptide. Oxidizing or reducing agents were omitted from experimental buffers to ensure a lack of indirect effects on KV1 conformation; previous observations indicate that oxidation state of ball peptides is preserved without presence of these agents in final buffers [8].

### Fourier Transform Infrared Spectroscopy

Peptide samples were loaded into a 10 µl volume gas-tight CaF_2_ cell (path length 6 µm). Infrared spectra were recorded using a 1750 *Perkin Elmer* FTIR spectrometer continuously purged with dry air. Samples were scanned 100 times per measurement in order to minimize noise, these 100 scans being signal averaged at a resolution of 4 cm^−1^. Double-sided interferograms were recorded and apodized using a raised cosine function prior to transformation of the data. Sample temperature was varied between 5°C and 80°C using a circulating water bath which facilitated control of sample temperature coupled with infrared absorbance measurements (taken every 5°C or 10°C as stated). The programs IRDM (*Perkin Elmer*) and GRAMS (*Galactic*) were used for data handling. Buffer spectra were recorded under identical conditions to their corresponding protein spectra and subtracted digitally to give a straight baseline in the 2100–1800 cm^−1^ area. Buffers prepared solely for buffer spectra subtraction measurements were prepared identically to their corresponding sample buffers except that SDS, DMPC, and LPC, which do not absorb in the amide I region of the infrared spectrum, were not included in the former. Second derivative spectra were calculated using GRAMS *Derivative* function with a 13 data point Savitzky-Golay smoothing window. Deconvolution of spectra was performed using GRAMS *Deconvolve (FSD)* function. Typically a full width at half height of 16 cm^−1^ and a Gamma factor of 3.5 were used with 60–75% Bessel smoothing. Curve-fitting of subtracted spectra was performed using GRAMS Curve Fit function, on the region from ∼1900 to ∼1550 cm^−1^, without fixing parameters, typically with 50 iterations.

### Interaction between ID and KV1 Peptides

Following reconstitution of KV1 into 100 mg/ml DMPC to a polypeptide concentration of 10 mg/ml, synthetic WT or V7E mutant ID peptides were added at room temperature, heated to 30°C to facilitate mixing, then returned to 20°C and allowed to equilibrate for several hours. Following this, temperature-resolved FTIR spectra were recorded. All ID- KV1 interaction experiments and controls were performed with the sample being held at a range of discrete temperatures between 5°C and 80°C, involving several heating and cooling cycles in order to judge reversibility or otherwise of conformational changes observed. WT or V7E ID peptides were mixed with KV1 polypeptide at molar ratios of 1∶2, 1∶1 or 2.5∶1 (ID:KV1) as indicated.

## Results

### Secondary Structure of KV1 Varies with Environment and Reconstitution Technique

First, KV1 polypeptide (10 mg/ml final concentration, as used throughout this study) was mixed with sodium dodecyl sulphate (SDS) to a final SDS concentration of 25 mM in DBS (pH 7.4), above the critical micellization concentration (CMC) for this detergent in these conditions. Simple mixing resulted in a predominantly aggregated peptide sample, characterized by cloudiness of the sample, and by a sharp intense peak at 1631 cm^−1^ in the amide I region of the infrared spectrum, with a minor amide I absorbance at 1657 cm^−1^ characteristic of α-helix (Fig. 1D) [20]. Using 25 mM SDS in DBS as a solvent, but solubilizing by the thin film method (no TFE), again resulted in a predominantly aggregated peptide, with a major infrared amide I absorbance at 1632 cm^−1^, and a secondary component arising from α-helix at 1654 cm^−1^ (Fig. 1E). KV1 prepared by thin film incorporation was, however, less aggregated than that prepared by simple mixing in SDS, as the contribution by α-helical structures in the infrared amide I region was proportionately greater using the thin film method.

Reconstitution by thin film method (no TFE) in 50 mg/ml LPC gave a predominant infrared amide I absorbance at 1654 cm^−1^, attributable to α-helical structures, and a secondary sharp peak at 1630 cm^−1^ arising from aggregated structures (Fig. 1F). Thus KV1 was more successfully incorporated into phospholipid micelles than into detergent micelles, but still with some nonspecific aggregation. Incorporation by thin film method (no TFE) into 25 mg/ml DMPC vesicles gave a similar infrared absorbance pattern (major 1654 cm^−1^ absorbance, secondary 1627 cm^−1^ absorbance – Fig. 1G). This indicated that phospholipid incorporation favored α-helix formation but was not 100% successful; furthermore it suggested incorporation into phospholipid vesicles (formed by DMPC) was favored over incorporation into micelles (SDS or LPC), as a smaller concentration of DMPC compared to LPC (25 compared to 50 mg/ml, respectively) was needed for comparable α-helix formation. DMPC was thus chosen for further reconstitution optimization.

### DMPC and TFE are Required for Complete Reconstitution of α-helical KV1

**Figure 2:**
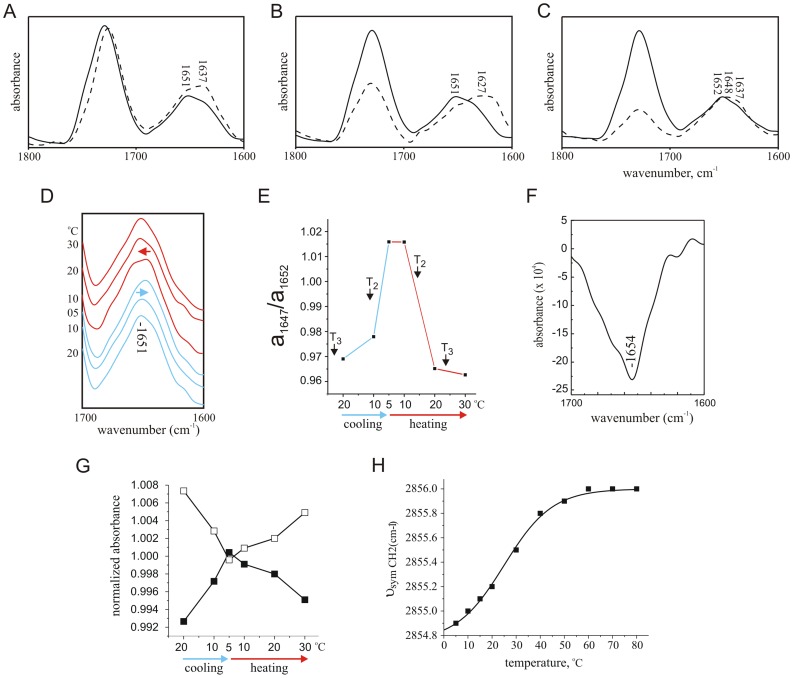
DMPC state-dependence of KV1 conformation. A. Deconvolved spectra of KV1 polypeptide (10 mg/ml) in 100 mg/ml DMPC with 10% TFE (dotted line) or 30% TFE (solid line) at 30°C. Peptide was prepared as a thin film, and then taken up into DMPC in TFE, before addition of deuterated PBS to give the appropriate final concentrations of TFE and DMPC. B. Deconvolved spectra of KV1 polypeptide (10 mg/ml) in 30% TFE with 50 mg/ml DMPC (dotted line) or 100 mg/ml DMPC (solid line) at 30°C. Peptide was prepared as in Panel *a*. C. Deconvolved spectra of KV1 polypeptide (10 mg/ml) in 40% TFE with 25 mg/ml DMPC (dotted line) or 100 mg/ml DMPC (solid line) at 30°C. Peptide was prepared as in Panel *a*. D. Amide I regions of deconvolved spectra of KV1 polypeptide (10 mg/ml) reconstituted by thin-film method into DMPC vesicles (100 mg/ml) in deuterated PBS with 40% v/v TFE at a range of temperatures during cooling from 20°C to 5°C (blue lines) and heating from 5°C to 30°C (red lines). The initial 1651 cm^−1^ absorbance peak is indicated; shifts in this initial peak are highlighted with arrows. E. Comparison of absorbance of infrared light at 1647 cm^−1^ relative to absorbance at 1652 cm^−1^, calculated from deconvoluted spectra, of KV1 polypeptide reconstituted by the thin-film method into DMPC vesicles (as in Panel *a*) at a range of temperatures during cooling from 20°C to 5°C (solid lines) and heating from 5°C to 30°C (dashed lines). DMPC phase transition points T_2_ and T_3_ are denoted next to their respective temperatures. F. Amide I region of KV1 polypeptide, 5°C minus 30°C difference spectrum, experimental conditions as in Panel D.G. Lipid phase transition - effect of decreased lipid hydration on the absorbance frequency of the ester carbonyl stretching band. Comparison of normalized absorbance of infrared light by DMPC at 1729 cm^−1^(open squares) and at 1730 cm^−1^ (filled squares), calculated from deconvolved spectra of DMPC (with KV1 polypeptide reconstituted by the thin-film method as in Panel *b*) at a range of temperatures during cooling from 20°C to 5°C and heating from 5°C to 30°C.H. Lipid phase transition - effect of gel-fluid transition on the absorbance frequency of the CH_2_ symmetrical stretching band. Absorbance frequency of the ∼2850 cm^−1^-centred lipid hydrocarbon chain CH_2_ symmetrical streching band was monitored with increasing temperature, from the absorbance spectra of DMPC (with KV1 polypeptide reconstituted by the thin-film method as in Panel b) at a range of temperatures during heating from 5°C to 80°C. The points were fitted with a standard Boltzmann function, giving values for A1 and A2 of 2854.8 cm^−1^ and 2856.0 cm^−1^ respectively, and a midpoint of 25.0±0.4°C for the DMPC gel-fluid transition (Chi^2^ = 4.2×10^4^).

Sample conditions were next optimized to prevent nonspecific KV1 aggregation. KV1 was incorporated by the TFE thin film method into varying concentrations of TFE and DMPC in DBS (pH 7.4) and the secondary structure content of KV1 at 30°C measured by FTIR spectroscopy. In 100 mg/ml DMPC, KV1 adopted a predominantly α-helical conformation (peak absorbance 1651 cm^−1^) with minor aggregation in 30% TFE (slight shoulder at 1635 cm^−1^), whilst in 10% TFE the amide I absorbance shifted to a dominant absorbance at 1637 cm^−1^ attributable to β-sheet, probably arising from sample aggregation (Fig. 2A). However, in 30% TFE, with the DMPC concentration reduced to 50 mg/ml, the predominant absorbance of the deconvolved infrared spectrum of KV1 shifted even lower in wavenumber to 1627 cm^−1^, attributable to strongly hydrogen-bonded β-sheet, highly characteristic of nonspecific aggregation [20] (Fig. 2B). In 40% TFE, KV1 in 25 mg/ml DMPC gave a peak at 1648 cm^−1^ attributable to mainly non-aggregated but also non-ordered structure, with a shoulder at 1637 cm^−1^ attributable to β-sheet; KV1 in 100 mg/ml DMPC (40% TFE), however, gave rise to a predominant peak at 1652 cm^−1^ (α-helix) with no shoulder in the 1630's cm^−1^ range (Fig. 2C). Lack of any significant absorbance between 1610 cm^−1^ and 1639 cm^−1^ under these latter conditions indicated an absence of non-specific aggregation, and no adoption of β-structure by KV1 [20]. Based upon previous studies examining effects of incorporation versus non-incorporation of similar TM peptides into lipid micelles and vesicles, we could thus conclude that KV1 had been successfully incorporated into the DMPC vesicles using the thin film technique, and that 40% TFE was also required to eliminate nonspecific KV1 aggregation [18], [20], [21]. Reconstitution conditions of 40% TFE, 100 mg/ml DMPC were therefore used for subsequent experiments.

### Helicity of DMPC Bilayer-incorporated KV1 Polypeptide Reversibly Reduces during the Lipid Liquid Crystalline-to-gel Transition

The temperature of the DMPC-held KV1 polypeptide was reduced to 5°C, below the T_3_ and T_2_ phase transitions of DMPC [22]. Infrared absorbance spectra were measured at steps during cooling from 20°C to 5°C and during subsequent heating to 30°C (above the T_3_ and T_2_ phase transitions of DMPC) (Fig. 2D). During cooling to 5°C, a reduction of the α-helical component of KV1 occurred concurrent with an increase in non-ordered structures, visualized as a shift in the major amide I absorbance from 1651 cm^−1^ to 1649 cm^−1^. Following this, heating to 30°C resulted in a shift of the major amide I absorbance to 1652 cm^−1^, indicating reversibility of the process and a return to a predominantly α-helical conformation upon re-heating (Fig. 2D, E).

Comparison of the relative intensities of the 30°C 1652 cm^−1^ (predominantly α-helical) absorbance and the absorbance at 1647 cm^−1^ (attributable to non-ordered structure) indicated a shift toward the latter upon cooling; the process was fully reversible upon reheating up to 30°C (Fig. 2E). Curve-fitting [23] (not shown) of the 30°C KV1/liquid crystalline DMPC spectrum yielded an estimate of 78±12% α-helix (1659±2 cm^−1^), with a smaller contribution (19±1%) by a 1655±0.3 cm^−1^ absorbance and a minor component (3±4%) at 1643±0.2 cm^−1^ attributable to non-ordered structures [24]. The broadness of the band at 1655 cm^−1^ was suggestive of contribution by mixed structures, but these were not separable by curve fitting without parameter fixing, which was avoided because of the artifacts inherent to this technique [23]. We therefore limit our assignation of the 1655 cm^−1^ curve-fitted band to ‘α_2_’: a mix of α-helix and/or non-ordered components, with the α-helix being perhaps less ordered than that absorbing at 1659 cm^−1^. Curve-fitting of the 5°C, KV1/gel phase DMPC spectrum yielded an estimate of 59±1% α-helix (1659±0.1 cm^−1^), 39±1% non-ordered (1644±0.1 cm^−1^) and 2±1% β-structures (1612±0.1 cm^−1^) in KV1. Subtraction of the 30°C spectrum from the 5°C spectrum, producing a difference spectrum, indicated that a net loss of α-helical contribution, absorbing at 1654 cm^−1^ (equivalent to the α_2_ component), occurred during the liquid crystalline-to-gel transition (Fig. 2F).

To study effects on the DMPC liquid crystalline-to-gel phase transition of the 40% TFE composition of the sample buffer, the state of the DMPC at various temperatures was monitored using infrared spectroscopy. Temperature-dependent shifts in hydration and polarity of the polar/apolar lipid interface can be monitored by examining the shifts in absorbance of the 1725–1735 cm^−1^ absorbance, which includes contribution from ester carbonyl stretching [25]. By monitoring absorbance intensities on either side of the initial peak, a shift to higher wavenumber corresponding to a reversible decrease in lipid hydration was visible upon cooling from 20°C to 5°C (Fig. 2G), as expected when the lipid adopts a closely-packed, quasihexagonal lattice structure at low temperature. A quantitative assessment of the DMPC gel-fluid transition was also possible by monitoring changes in the frequency of the CH_2_ symmetrical stretching band near 2850 cm^−1^ [25]. Measurement of the shift in infrared absorbance of this band between 5°C and 80°C showed a sigmoidal increase in wavenumber with increased temperature (Fig. 2H). Fitting these points with a standard Boltzmann function gave values for A1 and A2 of 2854.8 and 2856.0 respectively, and a midpoint of 25.0±0.4°C for the DMPC gel-fluid transition. This is in good agreement with the previous reported value of 22–24°C for the predominant transition in DMPC in aqueous conditions [22], thereby showing that the presence of 40% TFE in our sample buffer did not significantly perturb the DMPC gel-to-liquid crystalline transition.

### Synthetic K_V_3.4 ID Peptides are Non-ordered in DMPC/40% TFE at all Temperatures and Spectrally Silent at Concentrations Used in Interaction Studies

**Figure 3:**
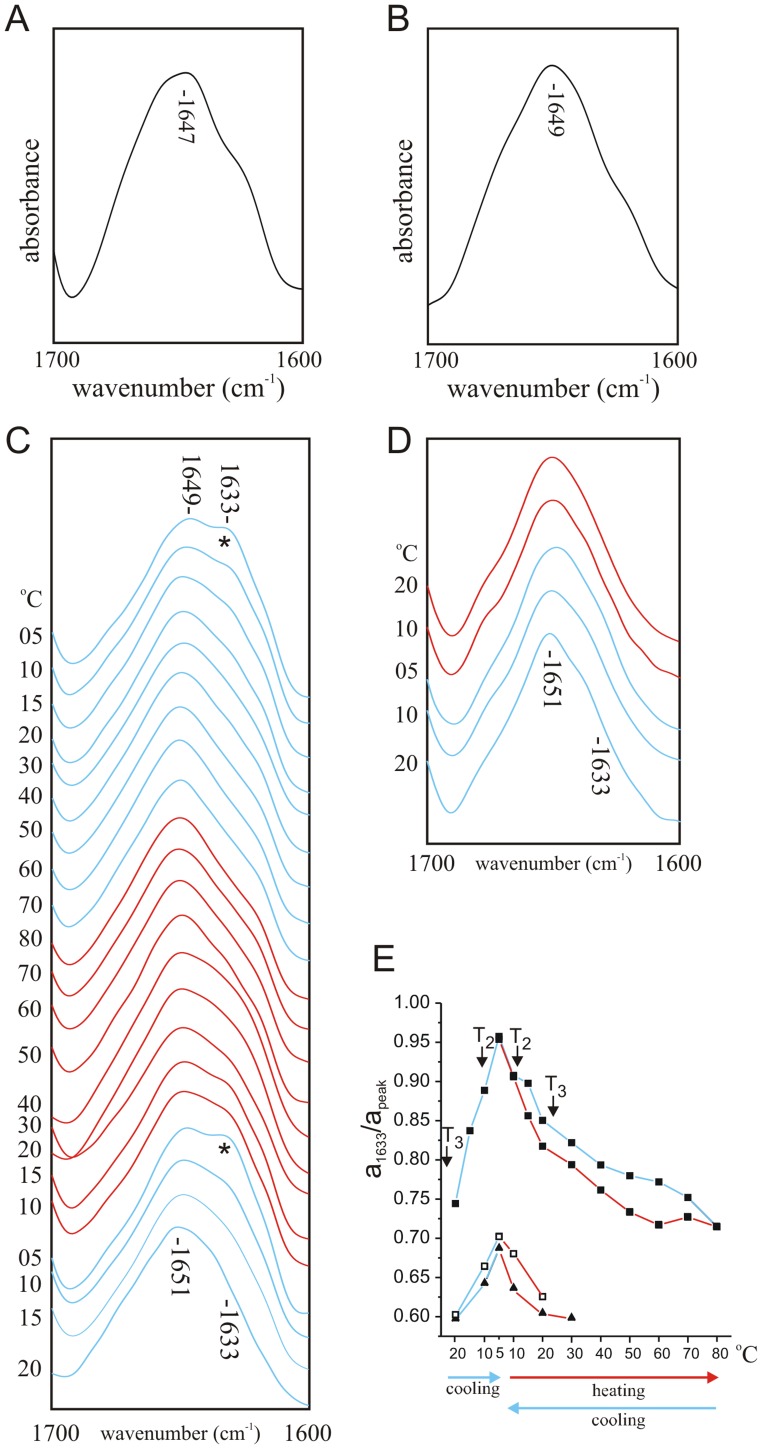
KV1-ID interaction monitored by FTIR. A. FTIR spectrum of wild-type ID peptide (10 mg/ml) at 5°C in deuterated PBS with 40% v/v TFE, 100 mg/ml DMPC. B. FTIR spectrum of V7E ID peptide (10 mg/ml) at 5°C in deuterated PBS with 40% v/v TFE, 100 mg/ml DMPC. C. Amide I regions of deconvolved spectra of KV1 polypeptide (10 mg/ml) reconstituted by thin-film method into DMPC vesicles (100 mg/ml) in the presence of 1 mg/ml wild-type ID peptide in deuterated PBS with 40% v/v TFE at a range of temperatures during cooling from 20°C to 5°C (blue lines), heating from 5°C to 80°C (red lines) and re-cooling from 80°C to 5°C (blue lines). The initial 1651 cm^−1^ absorbance peak and the emerging 1633 cm^−1^ peak (*) are indicated. D. Amide I regions of deconvolved spectra of KV1 polypeptide (10 mg/ml) reconstituted by thin-film method into DMPC vesicles (100 mg/ml) in the presence of 1 mg/ml V7E ID peptide in deuterated PBS with 40% v/v TFE at a range of temperatures during cooling from 20°C to 5°C (blue lines) and heating from 5°C to 30°C (red lines). The initial 1651 cm^−1^ absorbance peak and the 1633 cm^−1^ wavenumber are indicated. E. Comparison of absorbance of infrared light at 1633 cm^−1^ relative to absorbance at 1652 cm^−1^, calculated from deconvoluted spectra, of KV1 polypeptide reconstituted in DMPC vesicles at a range of temperatures during cooling from 20°C to 5°C (blue lines), heating from 5°C to 80°C (red lines) and re-cooling from 80°C to 5°C (blue lines); peaks from KV1+ wild-type ID peptide (filled squares) are compared with those from KV1+ V7E ID spectra (open squares) and from KV1 in the absence of ID peptide (filled triangles). Previously reported DMPC phase transition points T_2_ and T_3_ are denoted next to their respective temperatures.

FTIR spectroscopy was used to determine the conformation of WT and V7E forms of ID peptide, when solubilized in DMPC/TFE (40%) DBS buffer, at a peptide concentration of 10 mg/ml (tenfold higher than the concentration used for interactions studies, see below). The deconvolved FTIR spectra of neither WT nor V7E ID peptides altered significantly with changes in temperature. Spectra recorded at a sample temperature of 5°C show a single amide I absorbance at 1647 cm^−1^ for WT (Fig. 3A) and 1649 cm^−1^ for V7E (Fig. 3B). Thus, regardless of temperature, both IDs adopt a predominantly non-ordered conformation, as we previously observed for these peptides under aqueous conditions [8].

To determine the potential spectral contribution of the ID peptides when mixed with KV1, their infrared spectra at 1 mg/ml (the concentration used in interaction experiments) were assessed. Deconvolved subtraction spectra (not shown) of the ID peptides at this concentration showed no significant absorbance in the amide 1 region, indicating that the inherent amide absorbances of these low levels of ID peptide would not detectably contribute to spectra of the 10 mg/ml KV1 polypeptide with 1 mg/ml ID peptide added, amounting to a molar ratio of 2∶1.

### A Spectral Shift Consistent with Intermolecular Hydrogen Bonding upon Addition of ID to KV1

For interaction studies, ID and KV1 were mixed, then infrared spectra obtained during cooling from 20°C to 5°C and heating to 80°C, then re-cooling back to 5°C (Fig. 3C). A 1633 cm^−1^ band attributable to hydrogen bonding such as that occurring in β-sheet (42), not initially present at 20°C, emerged upon cooling to below 10°C. This 1633 cm^−1^ β-sheet band disappeared upon heating to 45–50°C, and reappeared upon re-cooling. A comparison of the intensity of the initial 1651 cm^−1^ peak to that of the emerging 1633 cm^−1^ peak in KV1+ ID spectra (▪), compared to the same wavenumbers in the absence of ID (Δ) clearly illustrated the requirement for ID peptide to produce the strong 1633 cm-1 absorbance (Fig. 3E). Curve fitting (not shown) of the 5°C KV1+ ID peptide spectrum revealed that KV1 now contained an estimated 73±1% α-helix (1656±0.2 cm^−1^), 19±0.5% unordered (1648±0.5 cm^−1^) and 8±0.5% β-structures (1629±0.4 cm^−1^; 1619±0.5 cm^−1^).

Similar experiments using higher concentrations of ID peptide (ID:KV1 molar ratios of 1∶1 and 2.5∶1 respectively) were performed. To eliminate any effects on the spectra arising directly from the increased ID content by virtue of its structure, spectra of buffer + ID at the appropriate concentration were subtracted from KV1+ ID spectra. These spectra did not significantly differ from those generated by the 1∶ 2 (ID : KV1) molar ratio, indicating no further induction of β-sheet with increased amounts of ID peptide (results not shown).

### V7E ID Peptide does not Cause Conformational Shifts in KV1 Polypeptide

Infrared spectra were next recorded for V7E ID mixed with KV1 at temperature increments when cooled from 20°C to 5°C (solid lines) and then heated from 5°C to 30°C (dashed lines). In contrast to findings with the “wild-type” ID (Fig. 3C), spectra recorded from V7E ID + KV1 (Fig. 3D) were not significantly different from those observed for KV1 in the absence of ID peptide (see Fig. 2), also evident from a quantification of the intensity of the 1633 cm^−1^ absorbance in KV1+ V7E ID spectra compared to those observed in the absence of ID, or with WT ID (Fig. 3E).

### Effects of Low Salt or Pre-oxidation on ID Interaction with KV1

The strength of electrostatic interactions within and between proteins can be altered by varying buffer salt concentration, although relationships can be complex and depend upon the type of protein and buffer ions used [26]. While increasing buffer ionic concentration often destabilizes electrostatic interactions because it provides competing ions for the charged residues involved, studies with the Greek key caspase recruitment domain [27], and cold shock protein CsP [28], indicated a loss of salt interactions at low ionic strength buffers. Similarly, increasing orthophosphate anion buffer ionic strength was previously found to increase thermal stability and activity of endooxylanase [29]. In general, it appears that structurally non-complex peptides such as the Kv3.4 ID, exhibit less pronounced buffer-defined electrostatic/binding effects than more structurally complex proteins [30]. However, as we suggest that the spectroscopic changes we observe upon the liquid to gel phase transition reflect intermolecular interaction of the ID with a larger, more complex protein (KV1), here we tested the effect of reducing buffer ionic strength on our spectroscopic shifts.

**Figure 4:**
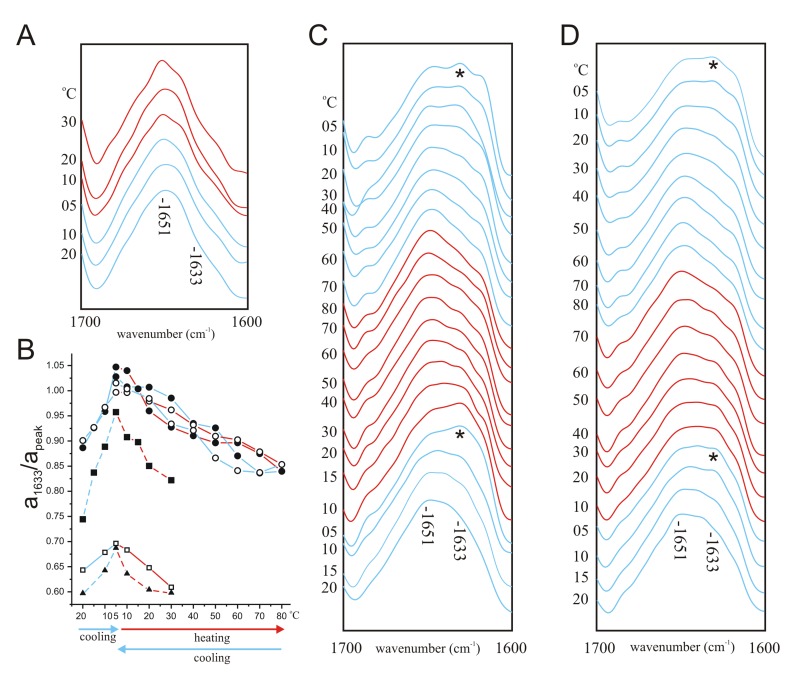
Effects of low salt and ID pre-oxidation on KV1-ID interaction. A. Amide I regions of deconvolved spectra of KV1 polypeptide (10 mg/ml) reconstituted by thin-film method into DMPC vesicles (100 mg/ml) in the presence of 1 mg/ml wild-type ID peptide in 1/10 deuterated PBS (‘low salt’) with 40% v/v TFE at a range of temperatures during cooling from 20°C to 5°C (blue lines) and heating from 5°C to 30°C (red lines). B. Comparison of absorbance of infrared light at 1633 cm^−1^ relative to absorbance at 1652 cm^−1^, calculated from deconvoluted spectra, of KV1 polypeptide reconstituted in DMPC vesicles at a range of temperatures during cooling from 20°C to 5°C (blue lines), heating from 5°C to up to 80°C (red lines) and re-cooling from to 5°C (blue lines), for the following: KV1+ wild-type ID peptide in low salt (open square); KV1 in DBS in the absence of ID peptide (triangle); KV1+ wild-type ID (filled square); KV1+ pre-oxidized wild-type ID (filled circle); KV1+ pre-oxidized wild-type ID in low salt (open circle). Dashed lines: values taken from earlier figures herein for comparison. C. Amide I regions of deconvolved spectra of KV1 polypeptide (10 mg/ml) reconstituted by thin-film method into DMPC vesicles (100 mg/ml) in the presence of pre-oxidized 1 mg/ml wild-type ID peptide in deuterated PBS with 40% v/v TFE at a range of temperatures during cooling from 20°C to 5°C (blue lines), heating from 5°C to 80°C (red lines) and re-cooling from 80°C to 5°C (blue lines). The initial 1651 cm^−1^ absorbance peak and the emerging 1633 cm^−1^ peak (*) are indicated. D. Amide I regions of deconvolved spectra of KV1 polypeptide (10 mg/ml) reconstituted by thin-film method into DMPC vesicles (100 mg/ml) in the presence of pre-oxidized 1 mg/ml wild-type ID peptide in 1/10 deuterated PBS (‘low salt’) with 40% v/v TFE at a range of temperatures during cooling from 20°C to 5°C (blue lines), heating from 5°C to 80°C (red lines) and re-cooling from 80°C to 5°C (blue lines). The initial 1651 cm^−1^ absorbance peak and the emerging 1633 cm^−1^ peak (*) are indicated.

The novel hydrogen bonding (amide I wavenumber 1633 cm^−1^) that emerged upon cooling the KV1-ID mixture was absent when using DBS diluted 1∶10 in D_2_O (Fig. 4A, B). The Kv3.4 ID contains cysteine residues at positions 6 and 24, which can be linked by disulfide bond with oxidizing agent to form a hairpin structure that is thought to enhance its inactivation properties [8]. Here, pre-oxidation of the ID to promote the hairpin structure increased the novel hydrogen-bonding that emerged upon cooling when ID was mixed with KV1 (Fig. B, C). Furthermore, pre-oxidation rescued the ability of ID to promote hydrogen bonding when mixed with KV1 in low-salt buffer (1/10 DBS) (Figure 4 B, D).

**Figure 5:**
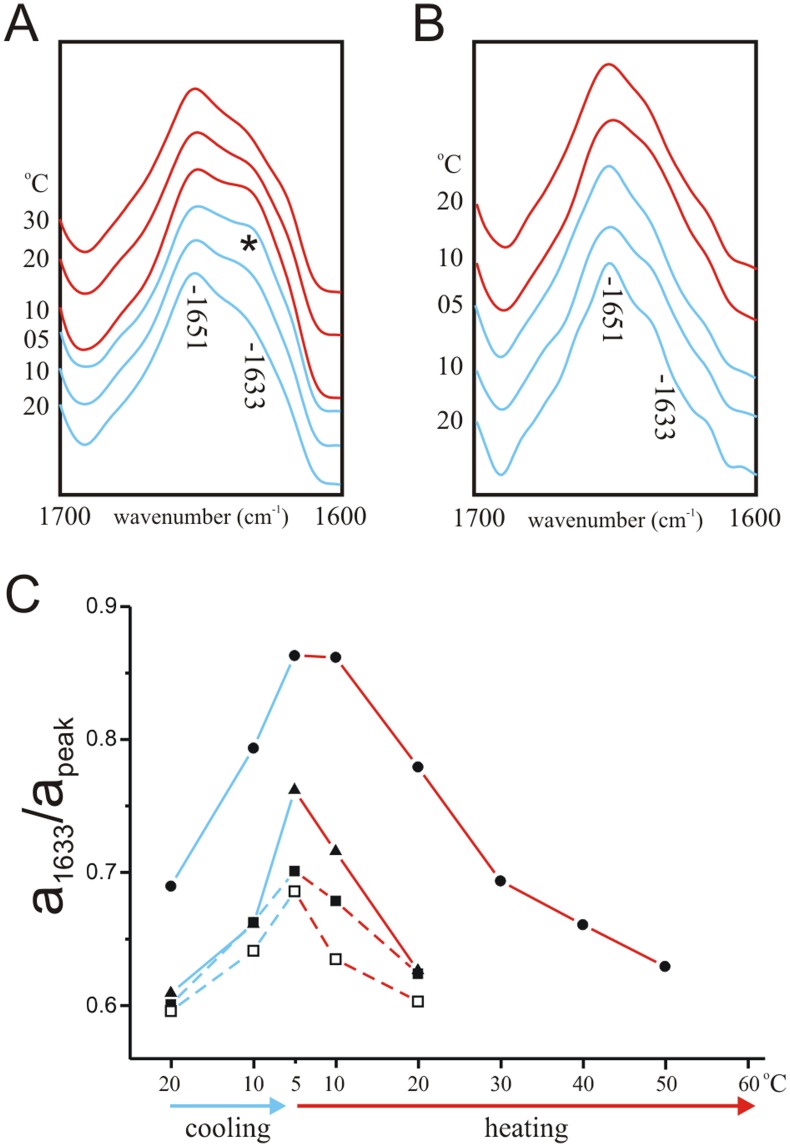
Effects of low salt and ID pre-oxidation on KV1- V7E ID interaction. A. Amide I regions of deconvolved spectra of KV1 polypeptide (10 mg/ml) reconstituted by thin-film method into DMPC vesicles (100 mg/ml) in the presence of pre-oxidized 1 mg/ml V7E ID peptide in deuterated PBS with 40% v/v TFE at a range of temperatures during cooling from 20°C to 5°C (blue lines) and heating from 5°C to 20°C (red lines). B. Amide I regions of deconvolved spectra of KV1 polypeptide (10 mg/ml) reconstituted by thin-film method into DMPC vesicles (100 mg/ml) in the presence of pre-oxidized 1 mg/ml V7E ID peptide in 1/10 deuterated PBS (‘low salt’) with 40% v/v TFE at a range of temperatures during cooling from 20°C to 5°C (blue lines) and heating from 5°C to 20°C (red lines). C. Comparison of absorbance of infrared light at 1633 cm^−1^ relative to absorbance at 1652 cm^−1^, calculated from deconvoluted spectra, of KV1 polypeptide reconstituted in DMPC vesicles at a range of temperatures during cooling from 20°C to 5°C (blue lines) and heating from 5°C to up to 50°C (red lines), for the following: KV1+ pre-oxidized V7E ID peptide in DBS (filled circle); KV1+ pre-oxidized V7E ID peptide in low salt (filled triangles); KV1+ V7E ID peptide in DBS (filled square); KV1 in DBS in the absence of ID peptide (open square). Dashed lines: values taken from earlier figures herein for comparison.

Pre-oxidation also rescued the ability of V7E ID to promote hydrogen bonding in normal salt conditions (DBS) (Fig. 5A, C) but the combination of the V7E mutation and low salt buffer (1/10 DBS) eliminated this rescue (Fig. 5B, C). In sum, the potent effects of ionic strength on the observed spectroscopic shifts were consistent with ID interaction with a structurally more complex protein, although the influence of pre-oxidation (disulfide bond formation within the ID hairpin) was sufficient to overcome this effect.

## Discussion

The ability to monitor interactions between proteins in real time and to simultaneously detect conformational changes in these proteins is highly desirable. Here, we present proof-of-principle that infrared spectral changes can be used to detect interaction between a soluble peptide and a phospholipid-embedded polypeptide. In this system, a previously identified conformational sensitivity of the phospholipid-embedded S4 to lipid phase change is exploited, permitting switching between interacting and non-interacting conformations - facilitating monitoring of reversible changes in infrared spectra dependent on presence of both peptides.

DMPC phospholipid undergoes a series of phase transitions dependent upon temperature [22]. The predominant, T_3_ transition occurs at 22–24°C and constitutes a hydrocarbon chain disorder-order transition (upon cooling) as the lamellar phase shifts to a more ordered low-temperature lipid phase. The T_2_ transition occurs upon cooling to below 11°C. Below 11°C, the hydrocarbon chains of the DMPC are fully extended and tilted with respect to the plane of the bilayer, but packed in a distorted, one-dimensional, quasihexagonal lattice. Cooling below the T_3_ and T_2_ transitions is associated with a decrease in the distance and therefore also the number of water molecules between each molecule of DMPC. Lattice formation in our system reversibly reduced the α-helical component of KV1. While the shift from α-helical to non-ordered structure we observe for KV1 could arise from any or several of the structural components of KV1, previous observations suggest that the most likely source of this spectral shift are those domains able to exist in non-aggregated form in aqueous solution - the S4 and/or S4–S5 linker. Infrared spectroscopy of a short synthetic peptide corresponding to the *Shaker* S4 domain alone, has demonstrated that it loses α-helical structure upon moving from lipid to aqueous solution [3]. In Kv channels, the S4 domain undergoes conformational changes in response to membrane depolarization which subsequently initiate channel opening. Based on a wealth of functional and more recently high-resolution structural data, these conformational changes are at present thought to constitute an outwards (intracellular to extracellular direction) movement of the entire voltage sensor “paddle” (S1–S4) with respect to other TM domains, or an actual extrusion of the S4 towards the extracellular space [13], [14], [31].

While we do not suggest that S4 extrusion in our model system closely mimics native S4 movement in response to depolarization, structural studies of isolated voltage sensor paddle indicate it forms a structure similar to that when attached to the pore module, and that it interact with phospholipid primarily via S4 and S3 (and less so with S1 and S2) [32]. Four lines of evidence are consistent with the hypothesis that the spectral shifts observed for Kv1 without ID in the current study, in response to the liquid crystal to get phase transition for DMPC, arise from conformational changes in the S4 domain and/or the S4–5 linker. First, the spectrum of the S4 domain alone in DMPC was previously found to contain a predominant absorbance at 1654 cm^−1^ [3]. The main amide I absorbance of 1654 cm^−1^ in the difference spectrum obtained from our data at 30°C and at 5°C for KV1 indicated a loss in the largely α-helical component absorbing at 1654 cm^−1^, corresponding well to the known absorbance of the S4 helix under these conditions (Fig. 2F). Second, curve-fitting of KV1 5°C and 30°C spectra indicated a reduction of the helical component by 20–40% concomitant with the DMPC bilayer shifting to gel phase (see Table 2). In KV1 polypeptide, 32% of the residues correspond to the S4 and S4–5 linker; the overall reduction was thus consistent with loss of helical structures largely in this region. Third, curve-fitting showed that the greatest reduction in α-helical content occurred in the 1655 cm^−1^ absorbance range, denoted the ‘α2' component in Table 2; again, this absorbance is similar to that previously reported for S4 [3]. Finally, the loss in α-helical component was reversible upon re-heating above the lipid phase transition temperature. This reversibility was consistent with previous spectroscopic studies on S4 movement which indicate reversibility and also a notable tolerance of the S4 TM domain for aqueous conditions [3], [33] and predictions that the S4-5 linker region of K^+^ channels (and the analogous stretch in Na^+^ channels) resides at least partially outside the membrane, adopting a partly α-helical, partly coil conformation [4], [13], [34]. A shift to outside the lipid environment of less hydrophilic portions of KV1 such as the S5 and S6 helices, would be expected to result in irreversible, non-specific aggregates, based upon previous observations [18], [21].

**Table 2:** Effects of lipid phase and inactivation ball peptide on secondary structure content of KV1 polypeptide.

experimental conditions	KV1 30°C	KV1 5°C	KV1+ WT ball 5°C
secondary structure content (%)	78 α; 19 α_2;_ 3 coil	59 α; 39 coil; 2 β	73 α; 19 coil; 8 β
lipid state	fluid	gel	gel

Values shown are percentage contribution of each secondary structure class as estimated by curve-fitting infrared spectra amide I regions. Curve fitting was performed in GRAMS (Galactic) typically with 50 iterations and no parameter fixing. ‘α_2_' in the KV1, 30°C column corresponds to a broad peak centred at 1655 cm^−1^, assigned to a mixture of α-helix and/or non-ordered structures.

### ID Peptide Initiates Hydrogen Bond Formation with KV1

Initial 20°C spectra of KV1 polypeptide with ID peptide added at a molar ratio of 2∶1 were no different from spectra of KV1 alone. Cooling to below 20°C, however, favored formation of some hydrogen bonding in the 1633 cm-1 range similar to that of β-sheet, which was reversible upon reheating; this correlated well with the disorder-order transition temperature of DMPC. The β-sheet component increased to a maximum upon cooling of the sample below the transition temperature of DMPC, and decreased accordingly when the sample was reheated above the DMPC transition temperature; these two effects only occurred in the presence of ID peptide, suggesting that conformational shifts in KV1 occurring concurrent with the DMPC phase transition exposed an ID binding site. Increasing the relative amounts of ball peptide to a molar ratio of 2.5∶ 1 (ball : KV1) did not result in any further adoption of β-sheet by KV1, inferring that the interaction was at a level maximal for the conditions with a 1∶ 2 molar ratio (ball : KV1). This is consistent with binding of the ID to a specific site, as nonspecific interaction between the ID and many regions of KV1, as might be expected for nonspecific aggregation between the two peptides, would result in greater amounts of β-structure with higher concentration of ball peptide, and would be irreversible.

Whilst inactivation ball peptides from different species have been demonstrated to adopt different conformations in aqueous solution, spectroscopic studies have shown that both the *Shaker* B and the K_V_3.4 inactivation ball peptides adopt a partial β-hairpin structure in a hydrophobic, negatively charged environment proposed to mimic the inactivation binding site of the ball [8], [11]. Mutation of the leucine at position 7 in the *Shaker* B ball peptide to a glutamate ablates the inactivation capability of the peptide [35] and also its ability to adopt β-structure [11]. Similarly, substitution by glutamate of the valine at position 7 in the K_V_3.4 inactivation ball peptide results in a loss of ability to attain β-structure [8]. Correspondingly, here we found that the V7E peptide (in reduced form) did not cause conformational changes in the KV1 polypeptide. A point mutation in the ID peptide was thus sufficient to eliminate interaction with KV1, again indicating a specific interaction, and one with requirements reminiscent of those for native ID channel inactivation [35].

### Assigning the Conformational Shifts Initiated by WT ID Binding

When considering the origin of the induced β-structure in our system, a minor component may have arisen from intramolecular hydrogen-bonding within the ID peptide, concurrent with hairpin formation. This would be consistent with ID-KV1 interaction facilitating adoption of the ‘inactivation conformation’ by the ID peptide. The 8% overall adoption of β-structure in the KV1/WT ball system, however, could not arise solely from the ID peptide, which was in low concentration and which still absorbs most strongly in the 1640–1650 cm^−1^ amide I region, even when in the ‘inactivating conformation’ [8]. Kv3.4 ID peptide does not contain periodic structures such as helix or β-strand when free in solution, but adopts a partial β-hairpin structure when in or near the proposed ball-binding site during channel inactivation [8], [36], [37]; accordingly, we observed formation of β-structure only when ID peptide was mixed with KV1. β-structure absorbing at 1633 cm^−1^ did not appear under the same conditions in the spectra generated by ID in the absence of KV1 even at a tenfold higher concentration than was used for KV1-ID interaction experiments (Fig. 3A), precluding the possibility that DMPC-ball interactions facilitated adoption of β-hairpin by the ball. Indeed, observations in previous studies have indicated that only insertion into negatively-charged lipids favors adoption of β-hairpin by WT ball peptide under standard conditions, and that the zwitterionic lipid DMPC would not cause such a change [8], [11].

Reversible intermolecular hydrogen bonding is thought to occur between the ball peptide and its pre-inactivation binding site on S4–S5. This type of interaction would be predicted to give rise to an amide I absorbance in the 1625–1639 cm^−1^ region, corresponding to formation of a specific, intermolecular β-strand complex [20]. Given that the amount of ID peptide used in our studies was spectrally silent, and that KV1 alone did not exhibit a 1633 cm^−1^ absorbance peak at any temperature, we consider it highly likely that the emergence of the 1633 cm^−1^ absorbance peak in ID-KV1 mixtures at temperatures below the liquid crystal to gel transition phase represents intermolecular hydrogen bonding between ID and a site on KV1, likely to be S4–S5. The findings that emergence of the 1633 cm^−1^ peak was prevented by the V7E mutation or by low salt conditions, but promoted by pre-oxidation of the ID peptide to favor hairpin formation via cysteine crosslinking within the ID, also support a specific ID interaction with S4–S5. Indeed, the ability of short synthetic peptides mimicking the ID and S4–S5 linker to interact was previously demonstrated using library screening [38].

In conclusion, our findings demonstrate proof-of-principle of the utility of FTIR in detecting and monitoring interaction between a soluble peptide and a membrane-embedded polypeptide. Future enhancements could include introduction of isotopic labeling to enable assignment of spectral shifts to specific components of this system, which would in particular assist when examining interactions of larger proteins with multiple domains. A further enhancement of the system could involve use of hydrogen-deuterium exchange to dynamically examine tertiary structural changes upon protein-protein interaction, which was not performed here to keep the number of temporally-variant factors low in the interests of simplicity and because we were examining reversibility. Finally, while we utilized synthetic proteins in this study, in future studies recombinant (and even purified native) proteins could easily be investigated. Use of scanning mutagenesis of recombinant proteins would also provide a cost-effective route to delineating more fully the requirement for specific residues in interaction processes including hydrogen bonding.
